# Short Message Service (SMS) Applications for Disease Prevention in Developing Countries

**DOI:** 10.2196/jmir.1823

**Published:** 2012-01-12

**Authors:** Carole Déglise, L. Suzanne Suggs, Peter Odermatt

**Affiliations:** ^1^Department of Epidemiology and Public HealthSwiss Tropical and Public Health InstituteBaselSwitzerland; ^2^University of BaselBaselSwitzerland; ^3^Institute of Public CommunicationFaculty of Communcation SciencesUniversità della Svizzera italianaLuganoSwitzerland

**Keywords:** Mobile health, developing countries, prevention, SMS, short message service

## Abstract

**Background:**

The last decade has witnessed unprecedented growth in the number of mobile phones in the developing world, thus linking millions of previously unconnected people. The ubiquity of mobile phones, which allow for short message service (SMS), provides new and innovative opportunities for disease prevention efforts.

**Objective:**

The aim of this review was to describe the characteristics and outcomes of SMS interventions for disease prevention in developing countries and provide recommendations for future work.

**Methods:**

A systematic search of peer-reviewed and gray literature was performed for papers published in English, French, and German before May 2011 that describe SMS applications for disease prevention in developing countries.

**Results:**

A total of 34 SMS applications were described, among which 5 had findings of an evaluation reported. The majority of SMS applications were pilot projects in various levels of sophistication; nearly all came from gray literature sources. Many applications were initiated by the project with modes of intervention varying between one-way or two-way communication, with or without incentives, and with educative games. Evaluated interventions were well accepted by the beneficiaries. The primary barriers identified were language, timing of messages, mobile network fluctuations, lack of financial incentives, data privacy, and mobile phone turnover.

**Conclusion:**

This review illustrates that while many SMS applications for disease prevention exist, few have been evaluated. The dearth of peer-reviewed studies and the limited evidence found in this systematic review highlight the need for high-quality efficacy studies examining behavioral, social, and economic outcomes of SMS applications and mobile phone interventions aimed to promote health in developing country contexts.

## Introduction

The last decade has seen an unprecedented growth of the development of information and communication technologies (ICT) infrastructure worldwide [1]. The trend exhibits a clear shift from landline toward mobile phones, whose subscription rates are three times greater than for landlines. The expansion has been most significant in the developing regions, where diffusion reached more than 40% of the population in 2007, thus connecting millions of previously unconnected people. Developing countries not only have the majority of world mobile phone subscribers, but will also account for 80% of the new ones [2].

 Mobile telephones offer short message service (SMS), also known as text messaging. SMS is a communication protocol standardized in the Global System for Mobile communications allowing messages of 160 characters maximum to be interchanged from a mobile phone or a computer to one or many mobile phones simultaneously [3]. SMS can send information in near-real time to thousands of people as recipients of standardized, bulk messages or even personalized or tailored messages. SMS is available on all cellular phones, including cheap low-end handsets, through the Global System for Mobile communications network.

Recent research has focused largely on the use of SMS for health purposes in developed countries, while compelling studies seem to remain anecdotal in the developing world [4-13]. But text messages could have considerable implications for disease prevention efforts in developing countries where their potential is recognized not only in their communication attributes, such as voice call and text messages, but also in their data transfer capabilities. In addition, the robustness, ease of maintenance, and relative affordability of handsets, compared with computers, make the mobile phone and SMS very attractive in resource-poor areas with little electricity and slow Internet connections. As such, an increasing number of mobile health initiatives are being implemented and tested in the developing world [14].

Primary and secondary prevention encompass all actions that help in “averting the occurrence of disease” and “halting the progression of a disease from its early unrecognized stage to a more severe one,” respectively [15]. As declared in the Ottawa Charter, health promotion is “the process of enabling people to increase control over, and to improve, their health. It supports personal and social development through providing information, education for health, and enhancing life skills” [16]. Today, the World Health Organization recognizes that “advanced information and telecommunication technologies should be employed to their fullest extent wherever possible, in order to create effective and transparent communication channels that will allow interactive sharing and learning among various groups of stakeholders in the society” [17].

With the rapid expansion of mobile health applications, combined with current and predicted economic and public health challenges, reviews of existing applications and evidence of SMS-supported interventions in developing countries are needed. Therefore, the objective of this study was to examine SMS-supported interventions for prevention of communicable and noncommunicable diseases in developing countries. We began with a review of peer-reviewed and gray literature on existing SMS-based interventions. Gray literature is “that which is produced on all levels of government, academics, business, and industry in print and electronic formats but which is not controlled by commercial publishers.” In other words, “grey literature includes documents that have not been formally published in a peer-reviewed format” [18]. Then, we assessed the effectiveness of SMS-based interventions and identified drivers and inhibitors to adoption. Finally, we present recommendations for future research and practice.

## Methods

The data used in this study are part of a larger review aiming to examine SMS-supported interventions for prevention, surveillance, management, and treatment compliance of communicable and noncommunicable diseases in developing countries. We used a subject-based approach to systematically search both peer-reviewed and gray literature published in English, French, and German up to May 2011. The decision to include gray literature was motivated by the fact that public health professionals often use gray literature to inform decision making at the practice, program, and policy levels [18], and that both gray and scholarly evidence should be included in reviews of new and innovative topics in order to reduce bias and provide good estimates of the effects of interventions [19].

### Data Sources and Search Strategy

Bibliographic database and search engines were PubMed (incorporating Medline), EMBASE, CINAHL, PsycINFO, ScienceDirect, SpringerLink, EBSCOhost, ABI/INFORM, Google Scholar, and Google. Synthesis-producing organizations comprised the Cochrane Collaboration, the Centre for Reviews and Dissemination, and the Campbell Collaboration.

 The search terms were *health promotion*, *public health*, *preventive health services*, *disease prevention*, *population surveillance*, *patient compliance*, *patient care management*, *disease management*, *self care*, *HIV*, *acquired immunodeficiency syndrome*, *tuberculosis*, *communicable diseases*, *communicable disease control*, *chronic disease*, *noncommunicable disease*, *cellular phone*, *mobile phone*, *cell phone*, *SMS*, *MMS*, text messag*, picture messag*, *developing countries*, *developing world*, *Asia*, *Africa*, *South America*, *Latin America*, and *Europe, Eastern*. We used the medical subject headings in Medline and Emtree tools in EMBASE. When necessary, these words were translated into French and German to minimize language bias.

The gray literature search strategy was inspired by a guideline for finding gray literature in public health [18]. Requests for cases and papers were posted on email lists, through personal connections, and directly to health organizations and SMS providers. Key gray literature sources were the Digital Repository Infrastructure Vision for European Research, the Electronic Theses Online System, the Networked Digital Library of Theses and Dissertations, the Directory of Open Access Repositories, DissOnline.de, SUDOC Catalogue, Social Science Research Network, Scirus, and Catholic Media Council Library. Other sources of gray literature comprised proceedings from related conferences, funding and not-for-profit organizations, listservs, blogs, SMS, and mobile phone providers. For identified projects, we contacted the corresponding agency to request documents and white papers that described the application and evaluation results. References of included documents and citation tracking of articles were also searched.

### Inclusion and Exclusion Criteria

Following a detailed protocol, an initial reviewer identified and retrieved all eligible documents. The titles and abstracts were first examined to remove obviously irrelevant reports. A second reviewer confirmed eligibility and relevance. The full text of the potentially relevant ones was reviewed to finally select eligible papers.

English-, French-, and German-language documents were included when they were related to interventions using SMS in developing countries for disease prevention. For the purpose of this review, SMS referred to all applications that used SMS or texting for disease prevention purposes. These included applications that automatically shifted between SMS and general packet radio service channels, had live-person components, and connected to the Internet. Developing countries were defined as *developing and emerging economies*, a World Economic Outlook classification system based on (1) per capita income level, (2) export diversification, and (3) degree of integration into the global financial system [20]. For this study, we considered countries classified as developing and emerging economies in the International Monetary Fund’s World Economic Outlook report in 2008 to be developing countries (eg, Algeria, Brazil, Cambodia, India, Mexico, Poland, and Zambia) [20].

Documents were excluded if the applications did not use SMS, if they were not used in developing countries, or when they did not focus on the prevention of communicable and noncommunicable diseases. Documents published in languages other than English, French, or German were also excluded.

### Data Collection and Management

Eligible articles were reviewed and data extraction was inspired by a template provided in the *Cochrane Handbook for Systematic Reviews of Interventions* [21]. Applications were given a unique identification number. Disease focus, country or countries of implementation, project objectives, target audience, status (planned, ongoing, or completed), funding source, and contact details were recorded. When an evaluation was reported, the methods used and outcomes measured (health outcome, process of care, relevance, acceptability, and cost effectiveness) were recorded. Each personal communication attempt was documented along with contact information and the outcome of the effort. Finally, SMS-based interventions used for health promotion and disease prevention were extracted from the larger dataset and used for this review.

## Results

The first screening identified 4008 citations. We retrieved 38 additional papers by personal communications (n = 21) and by hand searching the reference lists of eligible articles (n = 17). After an initial screening of abstracts and titles, we excluded most because they were related to developed countries or examined harmful effects of mobile phone use. A second selection was done to exclude applications that did not focus on disease prevention (eg, applications for surveillance, disease management, or compliance with a treatment).

**Figure 1:**
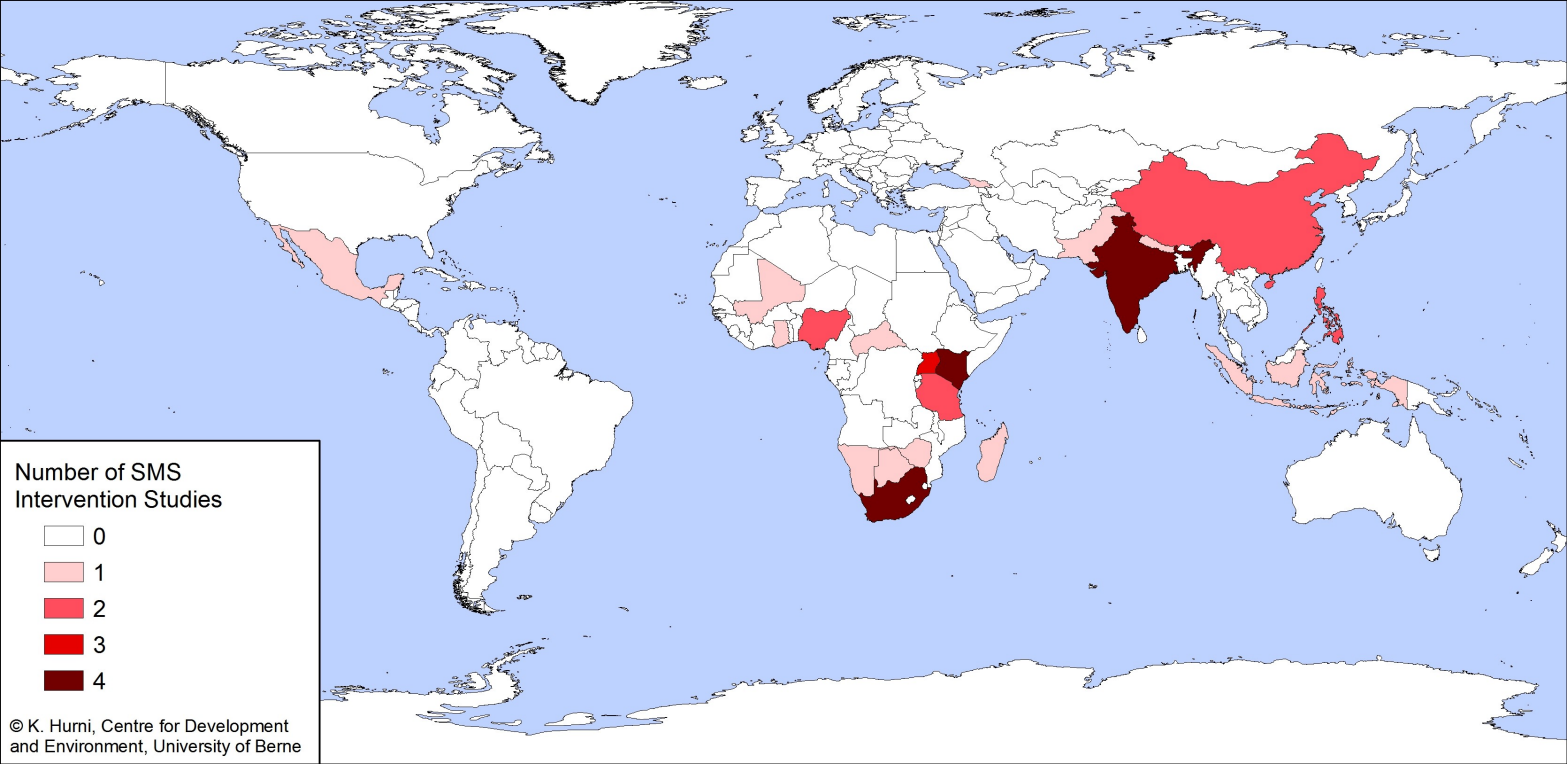
World map showing the distribution of short message service (SMS) intervention studies in developing countries (number of studies per country).

 We identified 34 different SMS-based prevention applications according to the eligibility criteria (Table 1 [22-67]), among which 5 included details about an evaluation (Table 2 [27,36,41,49-51,63]). Most were from gray literature sources. The interventions addressed a variety of topics, with human immunodeficiency virus/acquired immunodeficiency syndrome (HIV/AIDS) being the most common (n = 18), followed by sexual and reproductive health (SRH) (5). Some applications addressed multiple diseases (Table 3). Although projects extended across regions, SMS initiatives were concentrated in South Africa, Kenya, and India (Figure 1).

**Table 1:** Short message service (SMS) interventions for disease prevention

Intervention (reference)	Country	Disease	Description	Comments
It begins with you [22]	30 African countries	HIV/AIDS^a^	SMS voting system on what happened on the show, asked viewers to share what they have done to advance an HIV-free generation, and encouraged all Africans to start by knowing their HIV status.	Target: population; status: ended after 1 season
Star Project [23]	6 African countries	HIV/AIDS	Counterpart to India’s *Freedom from HIV* project. SMS used for downloading 2 mobile phone games (AIDS Fighter Pilot and AIDS Penalty Shoot Out) to raise HIV/AIDS awareness, deployed on low-end and sophisticated colored devices.	Target: population; technology: ZMQ; specificity: developed English and 2 local languages (Kiswahili and Shen)
Talk Back [24]	Botswana	HIV/AIDS	Weekly television program for HIV prevention, broadcasted live, to stimulate interactivity with teachers and viewers through phone lines, SMS, emails, and letters.	Target: teachers and students
UNICEF^b^ [25]	Central African Republic	Measles, malaria, diarrhea	Multimedia campaign used SMS to encourage vaccination, use of long-lasting insecticidal nets, and hand-washing.	Target: parents of young children
Text Me! Flash Me! [26,27]	Ghana	HIV/AIDS	Health education and promotion messages sent to mobile phone numbers collected by peer educators and social networks. Clients who text in “HELP” were referred to live helpline counselors, who called back within 24 hours.	Target group: most-at-risk populations: men who have sex with men and female sex workers
eQuest [28]	Kenya	HIV/AIDS	Contest engaged youth in discussions about HIV/AIDS. Youth sent SMS answers to questions about HIV/AIDS received on their mobile phone, after checking information in a special eQuest column printed in the newspaper.	Target: youth; incentives: airtime, T-shirts, mobile phone, computers, DVD players, and a home theatre system
Makutano Junction [29,30]	Kenya	HIV/AIDS	Soap opera based in a fictitious Kenyan village supported by SMS. Viewers were invited to text in if they needed more information on a given topic.	Target: population
Mobile4Good [31]	Kenya	HIV/AIDS	“My question” allowed customers to anonymously ask HIV/AIDS and breast cancer-related questions and receive answers via SMS. “Health Tips” provided subscribers with useful tips on various pertinent health issues via SMS.	Target: population
Afriafya [32]	Kenya	HIV/AIDS	Community resource centers worked with information and communication technology to access various information, including on health, via SMS request or other means of communication. Answer was sent back by email, booklet, or SMS.	Target: rural population; technology: telecenter
Pariah News [33]	Madagascar	HIV/AIDS	Citizen media-enabled project that broadcasted HIV/AIDS message via SMS, Internet radio, and blogs.	Target: sex workers; technology: Ushahidi platform, open source
Health On Line [34]	Mali	HIV/AIDS, malaria	Social marketing campaign that used bimonthly free SMS with health slogans and reference to an interactive sexual health website.	Target: young, urban people (n = 350,000)
Learning about Living [35,36]	Nigeria	HIV/AIDS and SRH^c^	Health promotion and prevention was based on HIV/AIDS, SRH, maternal morbidity, and gender violence with (1) MyQuestion: HIV/AIDS-related questions sent by public via SMS, Web, or hotline, answered by trained counselors, (2) MyAnswer: prizes won by texting correct answer to a quiz.	Target: young people; incentive: airtime; scaleup: in existing and new states
RapidSMS [37]	Nigeria	Malaria	SMS helped deploy bed nets by (1) tracking commodities from state stores to distribution points by monitoring coupon distribution, (2) sending SMS reminders about distribution times and location for beneficiaries.	Target: population; technology: RapidSMS (UNICEF innovation); license: open source
Beat It [38,39]	South Africa	HIV/AIDS	Free SMS to enter the draw for prizes that motivated people to check results on *Beat I* *t* television program. Designed to promote positive living, treatment access, and HIV infection prevention.	Target: youth; technology: Cell-Life; incentives: mobile phone, airtime
Cell-Life [40,41]	South Africa	HIV/AIDS	Mass messaging for prevention, linking clinic and patients to peer-to-peer support and counseling at no charge, through a computerized capture of mobile phone number and automatic SMS back with the information.	Target: patient; technology: Cell-Life; license: open source; multicomponent project; status: ongoing

Project Masiluleke [42,43]	South Africa	HIV/AIDS	Project provided several mobile phone-based applications for HIV/AIDS care: “Access Information” and “Get Tested”. Health promotion messages broadcasted in unused space of “Please Call Me,” a free form of SMS widely used in Africa.	Target: population; technology: SocialTxt from Praekelt Foundation; license: open source; multicomponent project compliance
South African Depression and Anxiety Group [44]	South Africa	Mental health	National toll-free suicide helpline and SMS for adolescents in crisis.	Target: young people; status: ongoing
Digital mosquito net vouchers [45]	Tanzania	Malaria	Implemented long-lasting insecticidal net distribution using SMS voucher system for controlling counterfeited voucher.	Target: pregnant women
Kimasomaso [46,47]	6 African countries	SRH	Radio program transmitted voices of young people keeping audio diaries, associated with helpline. Also provided SMS to redirect callers and text senders to local support.	Target: young people
AppLab [48]	Uganda	HIV/AIDS, SRH	Leveraged existing Village Shared Phone Operators to deliver mobile information services in health and agriculture with (1) SMS-based health tips and searchable database, (2) “Clinic Finder”, to locate nearby health clinics and services.	Target: population; technology: AppLab applications
Text to Change [49-51]	Uganda	HIV/AIDS	Interactive SMS quiz designed to help resolve key issues around HIV transmission and prevention, in the form of a multiple choice questionnaire that guaranteed free voluntary counseling and testing services to participants who answered correctly. Three quizzes offered weekly in English.	Target: population (15,000); incentives: voluntary counseling and testing services, airtime and mobile phone; status: ongoing, plan for Uganda and other African countries
UNICEF [52]	Zimbabwe	Cholera	Nationwide SMS information campaign during larger cholera campaign.	Target population
China Netcom [53]	China	SRH	SRH education and awareness campaign with SMS and hotline that gave access to medical experts.	Target: population and teenagers

SARS^d^ education [54,55]	China	SARS	Mobile phone subscribers could call an SMS that alerted them if they were within 1 km of a SARS-infected building, where confirmed cases existed, and about news updates.	Target: population; license: proprietary, mobile operator
Indonesia: Community Based Avian Influenza Control Project [56]	Indonesia	Avian influenza	SMS-based contest to encourage travelers in buses to be careful and to test their knowledge on the diseases.	Target: population; incentives: airtime
Condom Condom Campaign [57,58]	India	HIV/AIDS	Condom use promotion and HIV/AIDS awareness campaign among young men with (1) SMS opinion to vote on HIV/AIDS issues, (2) condom-themed mobile phone ringtone using SMS to get a push in reply, from where the user could download the ringtone.	Target: men; incentives: mobile phone and free talk time
Freedom HIV/AIDS [59]	India	HIV/AIDS	SMS used for (1) downloading mobile phone games to raise HIV/AIDS awareness, deployed on low-end and sophisticated colored devices, (2) announcement of radio shows on HIV/AIDS, (3) information on the nearest HIV testing center.	Target: population; technology: ZMQ; specificity: developed in local languages
Heroes Project [60,61]	India	HIV/AIDS	Multiple media channels including SMS to get key messages on HIV/AIDS out to the general public.	Target: population
Indian tuberculosis campaign [62]	India	Tuberculosis	Public awareness campaigns used SMS for tuberculosis information.	Target: population
Breast cancer awareness [63]	India	Cancer	SMS as reminder to conduct breast self-examination.	Target: working women in private companies
Global Hand-washing Day/UNICEF [64]	Nepal	Diarrhea	Public awareness campaign used SMS to encourage hand-washing.	Target: population
Mobilink [65]	Pakistan	Polio	Broadcasted millions of SMSs to encourage parents to get their children vaccinated against polio.	Target: parents; specificities: initiative of services provider
Sex-Ed Text [66]	Philippines	SRH	Computerized system using SMS to receive and then return the keyword of interest for getting complete and free information.	Target: young people
CardioNet [67]	Mexico	Cardiac diseases	Public prevention campaign in which users took a quick cardiac assessment screening by SMS.	Target: population; technology: Voxiva; license: proprietary

^a^Human Immunodeficiency Virus/acquired Immunodeficiency Syndrome.

^b^ United Nations Children’s Fund.

^c^ Sexual and Reproductive Health.

^d^ Severe acute respiratory syndrome.

**Table 2:** Short message service (SMS) interventions for disease prevention with an evaluation

Intervention	Disease	Aims	Methods	Results	Limitations/challenges
Learning About Living [36]	HIV/AIDS^a^ and SRH^b^	To document and distribute lessons learned during the process of initiating, planning, implementing, and monitoring the project.	Project evaluation; duration: 14 months after implementation; N = 9000 youth. Outcomes: objective and subjective.	User description: median age 24 years, 93% from urban and suburban settings, 79% male, 60,000 questions received by SMS, multiple use of service 49%. User satisfaction: 76%, 24% free, 12% prompt response, 7% easy access and availability, 24% educative HIV and SRH. Reason for dissatisfaction: >50% no answer due to bad network, 25% question partially answered, 18% response time too long, 7% question misunderstood.	Limitations: methods not described, results not comparable from state to state due to partner variation in evaluation methods. Challenges: network fluctuations, spam messages, girls and rural outreach.
Cell Phone for Life [41]	HIV/AIDS	To evaluate how the pilot service is perceived by the organizations running the service, those receiving the messages and those close to the recipients who may be affected: (1) baseline study of expectations of the pilot service, current access to information and service usage, and behavior, (2) process evaluation to assess how the pilot service is received, (3) review outcomes and results from the pilot service.	Baseline survey; N = 210. Outcomes: objective and subjective.	Participant characteristics: mean age 26.32 years, 71% female, 66.2% own a mobile phone, 79.5% comfortable using mobile phone, 64.3% use SMS. Qualitative information: Important: two-way communications important, messages may be consulted at any point, save time and money. Concerns: maintenance cost to members, the poor have no access to phones with required technology to perform Cell-Life functions technology, people change mobile phone regularly, potential for misuse or private use of SMS allocated by Cell-Life.	Challenges: maintenance and SMS costs, limited access for poor people, high mobile phone turnover, potential misuse or private use of SMS by Cell-Life.

Text Me! Flash Me! [27]	HIV/AIDS	To understand the main reasons for contacting the helpline.	Pilot project; duration: 6 months; N = 1169 calls; 12 health workers and 135 MSM^c^ randomly selected. Outcome: objective and subjective, interview and focus groups.	Participants: reach in first month: 5 counselors counseled 439 MSM clients = average of 88 MSM clients per counselor per month compared with 50 MSM clients per peer educator or health worker per month in facilities and communities; 87% shared the information with others: 40% did it by SMS to a mean of 8.6 persons; increased knowledge and intention to use condoms; 47% went to health services as counseled voluntary counseling and testing uptake increased after launch of campaign.	Challenges: lack of monetary incentives related by counselors.
Text to Change [49,50]	HIV/AIDS	Understand satisfaction with and use of pilot system.	Pilot project; duration: 8 weeks; N = 15,000. Outcome: objective, number of answers, and voluntary counseling and testing attendance.	Participants: 2610 actively participated, 807 texted back their age (mean 29.2 years), 801 texted back their gender (70.8% male; 29.2% female). Number of questions/participant: >1 (17.4%); voluntary counseling and testing attendance: 255 people.	Challenges: language barriers, confidentiality concerns (33.8%), lack of information on voluntary counseling and testing participants.
Text to Change [51]	HIV/AIDS	Assess access to and interest in receiving health information on mobile phone.	Survey; duration: 6 months. Outcome: number of answers.	Participants: 1506 with response rate of 86.7%; 62% male, 42% between 12 and 14 years and 51% between 15 and 17 years, 27% owned a mobile phone, among whom 93% sent SMS over past 12 months (34% every day, 35% weekly, 21% monthly, 9% <1/month), 19% of those who sent SMS said they did it to get health information in the last year; 51% of all adolescents said they were somewhat or extremely likely to access health education program through SMS, which was associated with owning a mobile phone; high-risk adolescents were equally likely to be interested in receiving HIV prevention program via SMS.	Limitations: low rate of mobile phone ownership in Mbarra, self-administered questionnaire, with results relevant to secondary school students with good English literacy. Large number of missing data.
Breast cancer awareness [63]	Cancer	To assess SMS effectiveness as reminders for making women aware of breast cancer.	Participants: 106 women; duration: 6 months; SMS to remind to conduct breast self-examination.	Participants: 20–54 years old. Outcome: among those who forgot, 54% forgot and intended to do it, 47% were busy, and 4% had some questions regarding the exam. After 2 months of reminders, the practice of breast self-examination improved significantly.	Limitations: small sample, little information on methods. Women working in private sector.

^a^ Human immunodeficiency virus/acquired immunodeficiency syndrome.

^b^ Sexual and reproductive health.

^c^ Men who have sex with men.

**Table 3:** Short message service (SMS) for disease prevention: disease focus of 34 applications

Disease focus	Number of applications	Percentage of sample
Human immunodeficiency virus/acquired immunodeficiency syndrome	18	47%
Sexual and reproductive health	5	13%
Malaria	4	11%
Diarrhea	2	5%
Others^a^	9	24%

^a^ Includes Measles, Avian Influenza, Cholera, Severe Acute Respiratory Syndrome, Tuberculosis, Poliomyelitis, Mental Health, Cancer, and Cardiac Disease. Some Applications Related to Several Diseases.

### Description of the Applications

The 34 applications, listed in Table 1 [22-67], were described using varying levels of detail about the purpose of the initiative and features. Most provided information about the method used for communication (one-way or two-way), the use of games and contests, and incentives to increase the adoption and use of the application.

#### One-way Communication

SMSs served as one-way communication tools for prevention. SMSs were sent outbound to large numbers of subscribers who had no opportunity to respond to messages or seek specific advice. The communication was standardized or targeted, tended to aim at a large population, and provided information about healthy behaviors and testing services. Such initiatives often required the participation of mobile phone operators who provided a database of phone numbers used during the campaign. As such, SMSs were sent with health promotion slogans for HIV/AIDS in the Heroes Project in India [60,61], to encourage parents to get their children vaccinated during Polio Vaccination Days in the Mobilink project in Pakistan [65], by the United Nations Children’s Fund for cholera information during an outbreak in Zimbabwe [52], to prevent diarrhea by encouraging hand-washing in Nepal [64], and to provide information about tuberculosis in India [62]. They were also sent to parents of young children encouraging vaccination, bed net use, and hand-washing in the Central African Republic [20]. For malaria prevention, text messages reminded the population about bed net distribution dates, times, and locations [37].

Cell Phone for Life in South Africa offered an open source system to support the fight against HIV/AIDS [40,41]. One component of the project diffused awareness through one-way mass messaging. The first stage of Project Masiluleke [42,43] circulated HIV/AIDS and tuberculosis messages to the general public. SMSs were sometimes accompanied by links to interactive website with, for example, information on sexual health for the project Health On Line in Mali [34]. In one component of Text Me! Flash Me!, SMSs were sent to subscribers with educational and promotional messages, either for a general communication strategy or in response to trends noticed through ongoing quality monitoring and evaluation [26,27].

#### Two-way Communication

Two-way communication interventions included opportunities for people to text in for health tips, obtain tailored information about clinic locations, or contact a live person. SMS also supported other media channels allowing people to ask questions when they needed more information. They could remain anonymous, which is particularly important for stigmatizing issues such as HIV/AIDS, other SRH issues, and tuberculosis.

 In the project Mobile for Good, “Health Tips” were sent to Kenyan subscribers who asked questions on HIV/AIDS and breast cancer-related issues [31]. An automatic computerized system was also established in the Philippines. In the Sex-Ed Text project, people who texted “SET” to a specific number (ie, short code) received a menu of keywords for SRH. When they responded with the keyword of interest they received free information [66]. Some projects leveraged existing public access services to deliver the intervention. Launched in Uganda, the Application Laboratory used shared phone operators to deliver information through an SMS-searchable database of health tips on sexually transmitted diseases, family planning, and maternal health, as well as details on local clinics and timetable of operation for outreach services [48]. In China, individuals could text in to receive the locations of buildings affected by severe acute respiratory syndrome and know of cases within 1 km of the calling location [54,55]. Users could also text in for daily updates on the syndrome. Messages were in both English and Chinese.

Several initiatives used television and radio programs in combination with SMS to engage in two-way communication. In the television soap opera *Makutano Junction*, viewers were encouraged to send an SMS if they needed more information about HIV/AIDS [29,30]. During the first, year, 30,000 text messages were received, and audience research showed that such a medium was useful to deliver information to rural and periurban audiences. Similarly, the It Begins With You campaign used SMS to engage young people in watching a show and voting with SMS [22]. The campaign aimed to get young people to share what they are doing to advance an AIDS-free generation and to know their HIV status. The Botswana Talk Back initiative aimed to provide education about HIV and involved a television talk show where viewers were encouraged to SMS with the presenters in the studio during live broadcasts [24]. *Kimasomaso* was a radio series that provided education about SRH issues [46,47]. Individuals could send in a text and receive support and advice.

Another component of the previously mentioned Cell-Life project used two-way communication to provide information on the location of voluntary counseling and testing (VCT) clinics [40,41]. Anybody could dial a specific phone number associated with a certain piece of information. A computer captured the number and sent an SMS back with the requested information at no charge to the requester. In Kenya, Afriafya provided community resource centers with technology access to HIV information [32]. Individuals requested information through SMS, and information was returned by SMS, email, or in print. Pariah News in Madagascar used SMS to send news about HIV/AIDS testing and prevention to sex workers [33]. Sex workers updated their health and HIV status and reported cases of violence anonymously, willingly, and in real time.

The Project Masiluleke connected mobile users to existing call centers, where trained operators provided health information, counseling, and referrals to local testing clinics [42]. After 3 weeks, average daily call volume to the national AIDS helpline tripled in Johannesburg, South Africa. Another component of the HIV/AIDS education initiative Text Me! Flash Me! in Ghana allowed for two-way communication. Targeting men who have sex with men and sex workers, users could text HELP to the project shortcode and a live peer counselor called back within 24 hours [26]. The South African Depression and Anxiety Group provided a national toll-free suicide crisis line and an SMS for adolescents in crisis [44]. In Tanzania, pregnant women could receive digital vouchers for bed nets, to prevent malaria [45]. Doctors sent an SMS to a retail bed net outlet and received back a valid digital voucher code. That code was transferred to the woman, who could then pick up a bed net. This also reduced the spread of fraudulent paper vouchers. In China, subscribers of two local mobile providers could text with experts about SRH issues [53].

Another project evaluated the use of SMS on breast cancer prevention, targeting working women in the private sector in Delhi. An SMS was sent every month according to the person’s last menstrual cycle to remind her to conduct breast self-examination at the end of her menstrual period. The content not only reminded the women to conduct breast self-examination, but also asked them to send an SMS back stating whether they had done it [63].

#### Games and Contests

**Figure 2:**
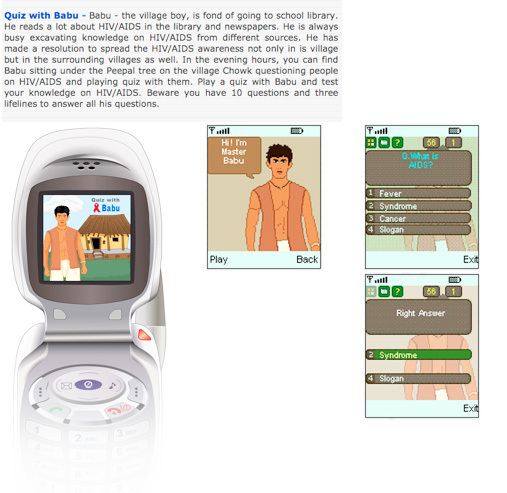
Freedom HIV/AIDS game: Quiz with Babu. Reproduced with permission [59].

The Indian Freedom HIV/AIDS project [59] and its African counterpart, the Star Programme [23], both used games to increase participation and promote disease prevention behaviors (Figure 2 [59]). Both used mobile platforms that were deployable on low-end black-and-white as well as sophisticated color cell phones to purposely target different preferences and socioeconomic groups. In India, 10 million game sessions were played over a 15-month period, and in Africa 6 million games were played in the first year. The project later included other topics such as tuberculosis, malaria, swine flu, lifestyle, and women’s health issues.

 The Condom Condom Campaign in India used different approaches to encourage young men to communicate about HIV/AIDS and to use condoms [57,58]. An SMS opinion vote was published in print media around the core message “smart men talk about condoms.” In six newspapers across four states, an average of 89% of readers responded to a text vote. In addition, to get people to talk about condoms, the project developed a condom-themed ringtone that people could download via SMS shortcodes (ie, dedicated 5-digit mobile phone numbers). There were 270,000 requests for it.

Other issues addressed by games and contests included avian flu and heart disease. In 2005, the Community Based Avian Influenza Control Project in Indonesia, an SMS-based contest, encouraged travelers in buses to test their knowledge of Avian Influenza [56]. In Mexico, CardioNet used a quick cardiovascular risk assessment screening by SMS [67]. In turn, the numbers collected were used to send advice and information about heart health. The company was also developing a comparable tool for breast cancer information.

### Incentives

**Figure 3:**
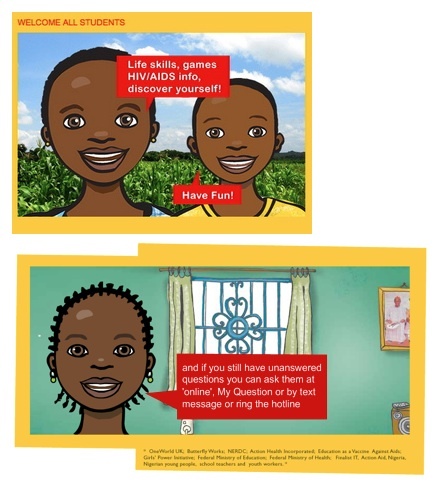
Learning about Living. Reproduced with permission [35,68].

Some projects generated participation with a rewards system. Text To Change in Uganda designed an SMS multiple choice quiz, three times per week, to raise HIV/AIDS awareness and increase VCT [45]. Parallel to radio and newspaper advertisements, background information was provided in a special column in a magazine. Users submitting correct answers to the quiz were offered free VCT services and the possibility to win mobile phones or airtime. The program also monitored knowledge and beliefs through responses. The project Beat It in South Africa used free SMS to enter a prize draw where the results were announced during a television show designed to promote positive living and HIV/AIDS prevention [38,39]. This drew viewers and provided a contactable database. Campaigns such as Learning about Living in Nigeria (Figure 3) [35,36,68] and eQuest in Kenya [28] often targeted youth, encouraging them to correctly answer questions on HIV/AIDS issues for free airtime, T-shirts, mobile phones, computers, or DVD players.

### Evaluations and Outcomes

Most of the reported applications did not provide information about an evaluation. Five interventions reported outcomes, with varying levels of detail (Table 2 [26,27,36,41,49-51,63]): Learning About Living [36], Cell for Life [41], Text Me! Flash Me! [27], Text to Change [49-51], and Breast Cancer Awareness project [63]. Each of these is described below including available information about the aim and method used to evaluate it. Findings are described, and stated benefits and limitations are then noted.

The project Learning about Living, an HIV and SRH education campaign, was evaluated after 14 months of implementation [36]. The project reached 9000 young people (median age of 24 years) who submitted 60,000 HIV/AIDS-related questions by SMS, and 4500 people (50% of users) accessed the service more than once. Most users (93%) were from urban or suburban setting and were male (79%). The majority (76%) were satisfied with the service, mostly because it was free (24%), quick (12%), or easily available (7%). Among dissatisfied users (24%), half did not receive a response to their SMS due to mobile network fluctuation, and 50% complained about the timing of answers. Despite the lack of information on methodology and variation in methods of evaluation between different sites, this initiative may have increased access to information for young people. The strengths of the project were regular formal and informal communication between all stakeholders. Noted challenges included network fluctuations, spam issues, and low reach of women and rural populations.

The pilot service of Cell-Life was evaluated using quantitative information from 210 respondents as well as qualitative inputs from 10 in-depth interviews and 5 focus groups [41]. Among the 210 participants, 66% owned a mobile phone, 80% felt comfortable using a mobile phone, and 64% were comfortable using SMS. Awareness of Cell-Life was poor, and its primary role was perceived as information dissemination at multiple levels. Users ranked the two-way communication and 24-hour availability of the messages for consultation on the mobile phone as important aspects, with the principle advantage that Cell-Life would save them time and money. Many requests were received for information about HIV and health in general, medication reminders, clinic locators, and access to reference sources of experts. Barriers included concerns about maintenance and SMS costs, limited access for poor people, high mobile phone turnover, and potential misuse or private use of SMS.

 The Text Me! Flash Me! Service was an HIV/AIDS education initiative in Ghana that strived to strengthen HIV/AIDS knowledge among the most-at-risk population. After 6 months of implementation, the initiative was evaluated [27]. The aim was to understand the main reasons for using the service. Participants reported sharing information they received with others (87%) and 40% of them did so by SMS. The most popular service of the system was referral to VCT services (71% of the calls), which resulted in a substantial increase in VCT uptakes. Traditionally, counseling occurred at a rate of 50 clients per week per counselor. This program resulted in each counselor providing advice for approximately 88 clients per week. Reported challenges included a lack of financial incentives for counselors.

 The project Text to Change aimed to increase the use of VCT centers. The system was evaluated in several papers, including 1 published in peer-reviewed literature [49-51]. In 2 papers, the project showed a 40% increase of VCT uptake after using an SMS quiz [49,50]. Identified challenges included language barriers due to English-language messages, a majority of male participants, confidentiality concerns (33.8%), and missing data (no information collected at VCT centers). The low number of respondents highlighted the importance of marketing at the beginning of the campaign and reassuring anonymity. In the quantitative study published in 2011 [51], the reported response rate was higher (86.7%) and still characterized by a majority of male participants. Among the SMS users, 19% mentioned they used it to get health information, and slightly more than half (51%) said they would be extremely likely to access health education programs through SMS, which was correlated with owning a mobile phone. High-risk and low-risk adolescents were equally likely to be interested in receiving HIV prevention programs via SMS. This study suggested that not all adolescents would have access to a mobile phone intervention and that nearly half would not necessarily choose to receive HIV/AIDS health information via this channel. It shows that adolescents may engage with education in different ways, thus emphasizing the importance of making HIV/AIDS prevention messages available in different modes and environments.

In India, the community-based study to evaluate the effect of SMS on breast cancer prevention targeting working women in the private sector in Delhi reported a significant increase of women performing breast self-examination after 2 months of SMS reminders [63]. The study sample was small and targeted only highly educated women working in the private sector who own mobile phones.

## Discussion

This review collected descriptive information about SMS-based disease prevention interventions in developing countries, reported as of May 2011. Applications were widespread with small clusters in South Africa, Kenya, and India. Most of them targeted HIV/AIDS and SRH, but the topics were broad. Many SMS-delivered applications were initiated by projects with modes of intervention varying between one-way and two-way communication. Some used educative games or incentives to increase participation.

Of the 34 applications, 5 reported an evaluation. The evaluations primarily focused on the processes and outputs of the interventions. However, some provided evidence on behavior change outcomes, such as increase in uptake of VCT services and increase in breast self-examination. Available data suggest that for the 5 initiatives evaluated, SMS interventions were feasible, were well accepted by the targeted population, and allowed for high reach. Additionally, several projects that did not conduct or report an evaluation did report usage statistics that suggest the target audience used the service (at varying levels of engagement).

Some issues appeared as potential barriers to usage. These included lack of timely responses, mobile network connection fluctuations, lack of financial incentives, maintenance and SMS costs, high mobile phone turnover, and potential misuse or private use of SMS. Language barriers were identified as limiting factors in one campaign [49,50]. While some countries such as South Africa have 11 official languages and many more indigenous ones [69], scalability of the intervention may depend on the ability to develop health promotion messages in various languages and ethnocultural contexts, and finding ways to target people speaking less-known languages. A key learning of the consultation was that ICTs are “only as good as the information they seek to communicate” and the importance of considering “the whole communication process of which ICTs have opened up unprecedented opportunities.” [5] It is important to develop culturally and gender-sensitive messages and approaches provided in the languages of the target population. Transmitting messages through intermediaries, developing a voice recognition system, or using pictograms or graphs may be worthwhile solutions to language barriers. The limited length of SMS (160 characters) also influences content; however, next-generation mobile services will allow the transmission of richer messages and multimedia capacities, affording new opportunities for more effective communication.

There were also concerns about maintenance and SMS costs, high mobile phone turnover, and reliability and security of systems. Reliability may be questioned by the sender, who does not know whether the messages have been delivered, and by the receiver, who may not receive the message [70]. This was of particular importance with the high mobile phone turnover reported [41]. Users raised concerns about the ability to ensure data protection and confidentiality [49,50], even though the mobile phone was perceived as the most trustworthy technology over others [70]. These aspects were particularly important in settings where users shared mobile phones. It has been an undeniable concern for patients with tuberculosis and HIV/AIDS, who would have increased risk of stigmatization if their text message were viewed inadvertently, unless the message was encrypted. This was particularly of concern in South Africa, where health workers were repeatedly robbed at gunpoint for their mobile phones while making their home care visits [71].

Finally, as an unintended consequence of the high reach afforded and achieved using SMS, health workers may bear the larger burden of servicing a larger number of clients than before, creating problems in quality or in feelings of not being properly compensated [27]. This may be a challenge to programs that do not have the resources to enhance financial incentives of workers. Thus, careful planning about capacity of systems and their staff must be fully assessed prior to launching SMS initiatives that aim to increase use of services, addressing the potential increase in the staff workload.

Insights provided in this review outlined current practice of SMS for disease prevention in developing countries. Novel projects were doing innovative things to prevent the spread or progression of disease. Using games and interactive communication strategies, as well as integrating SMS with other channels, is a reality and holds great promise for reach and potential social change. Yet high-quality SMS-based intervention studies from developing countries were lacking in the literature, as reported in other recent literature reviews [72,73]. Most of the evaluations in this study were feasibility studies assessing process and output, rather than outcomes, a situation that has been called the pilot syndrome [74]. The included case studies predominantly appraised process and usage output and encompassed various limitations, such as (1) sample selection (no randomization; often convenience samples), (2) sample size (insufficient statistical power), (3) lack of information on process validations (pretesting, recruitment type, response rate, retention rate), (4) lack of assessment of the maintenance of the effect, and (5) no comparison with control groups. Given the enormous potential of this widespread communication tool, randomized controlled trials to determine efficacy of using SMS as a means for disease prevention in developing countries are highly needed and should be a priority in funding.

Limitations related to the literature review process should be considered when interpreting the results. The innovative aspects and the commercial implications of the field may have affected the reporting of studies. The findings could be influenced by a tendency of projects to report positive results, by time lag issues typical of a fast-moving field, by projects promoted by industry, or by unwillingness to share information in order to protect innovation. Additionally, this review includes SMS applications focused on disease prevention and thus does not provide a full picture of applications with other foci, such as disease surveillance or adherence to prescribed regimens. Results may furthermore have been influenced by the involuntary omission of documents from the gray literature, which were sometimes difficult to find [18]. The extensive search and the methodical approach used in this review strived to minimize limitations and enabled the assembly of comprehensive information about this innovative field.

### Conclusion

There are many SMS health initiatives for disease prevention in developing countries, yet few are being evaluated and reported. Those that did conduct evaluations reported process evaluation and uptake, providing limited data about behavior change. Moreover, with a low number of documents found in the peer-reviewed literature, it appears that, to date, little is being done to advance our understanding of what works and what outcomes could be achieved in using SMS for disease prevention in a developing country contexts. Major opportunities are perceived, evident by the number and wide variety of projects, the recent creation of the United Nations Foundation’s mHealth Alliance, and papers published describing SMS applications in the developed world [6-14,75]. However, the need remains for evidence-based dissemination of information about using mobile phones and SMS for improving health in the developing world. The limited evidence found in this systematic review highlights the need for research that assesses behavioral, social, economic, and health outcomes of mobile phone interventions aimed at promoting health in developing country contexts. Sharing best practices and providing strategic directions will allow the building of evidence for the use of mobile health technologies that address the needs of health systems, communities, and populations. Consequently, the promises of using mobile phones and SMS may translate into an equitable improvement in health.

## Abbreviations

HIV/AIDS: human immunodeficiency virus/acquired immunodeficiency syndrome
ICT: information and communication technologies
SMS: short message service
SRH: sexual and reproductive health
VCT: voluntary counseling and testing
